# Early gastric adenocarcinoma with enteroblastic differentiation diagnosed synchronously with a conventional gastric adenocarcinoma

**DOI:** 10.1055/a-2443-3937

**Published:** 2024-11-13

**Authors:** Xiaonan Shen, Heng Zhang, Zhengting Wang, Xi Chen, Aihua Qian

**Affiliations:** 1 66281 Department of Gastroenterology, Shanghai Jiao Tong University Medical School Affiliated Ruijin Hospital, Shanghai, China; 2 66281 Department of Pathology, Shanghai Jiao Tong University Medical School Affiliated Ruijin Hospital, Shanghai, China

A 59-year old man underwent an esophagogastroduodenoscopy (EGD) for upper abdominal discomfort, which showed two different lesions: lesion 1 was a 0-IIa, slightly reddish, 15×8-mm lesion in the gastric angle; lesion 2 was a 0-IIc, reddish, 8×8-mm lesion in the cardia ( Fig. 1 ). On narrow-band imaging (NBI), the 0-IIa lesion appeared slightly brownish, whereas the 0-IIc lesion appeared dark brownish. The demarcation line of 0-IIa lesion was less clear than that of the 0-IIc lesion. On magnifying NBI, the 0-IIa lesion presented an irregular microvascular pattern with loop-like structure. Within the irregular microsurface pattern, the intervening part was wide and elongated ( Fig. 2 ). The irregular microvascular and microsurface patterns of the 0-IIc lesion presented loop and irregular mesh patterns with fused glands, an unclear white zone, and white globe appearance ( Fig. 3 ). In brief, the 0-IIa lesion seemed to be behaving with a higher degree of differentiation than the 0-IIc lesion.

**Fig. 1:**
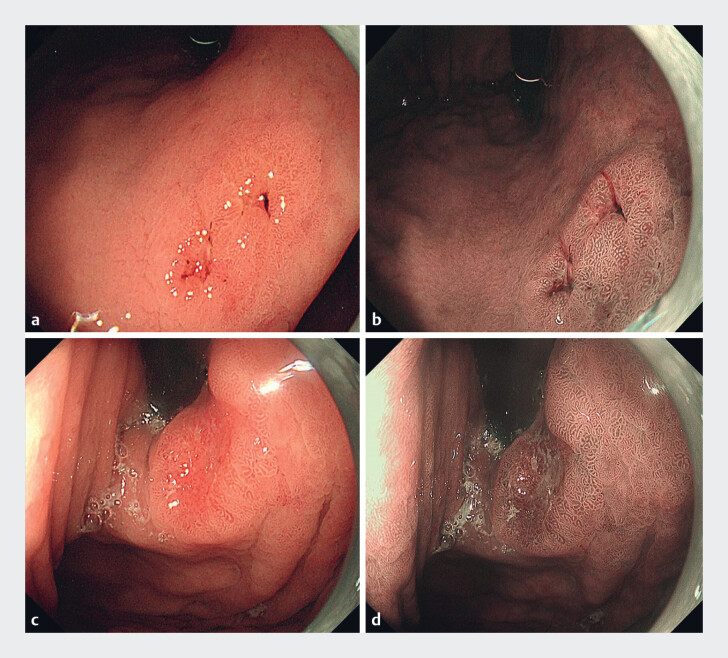
Endoscopic images of: **a, b** the 0-IIa lesion in the gastric angle showing: **a** on white-light imaging (WLI), a slightly reddish lesion of 15×8 mm; **b** on narrow-band imaging (NBI), slightly brownish coloration; **c, d** the 0-IIc lesion in the cardia showing: **c** on WLI, a reddish lesion of 8×8 mm; **d** on NBI, brownish coloration.

**Fig. 2:**
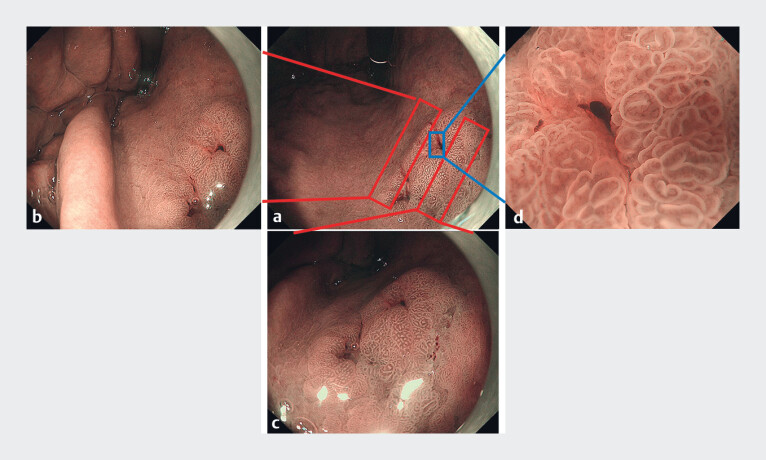
Detailed images of the 0-IIa lesion showing a clear demarcation line, irregular microvascular pattern with loop pattern, irregular microsurface pattern with wide and elongated intervening part.

**Fig. 3:**
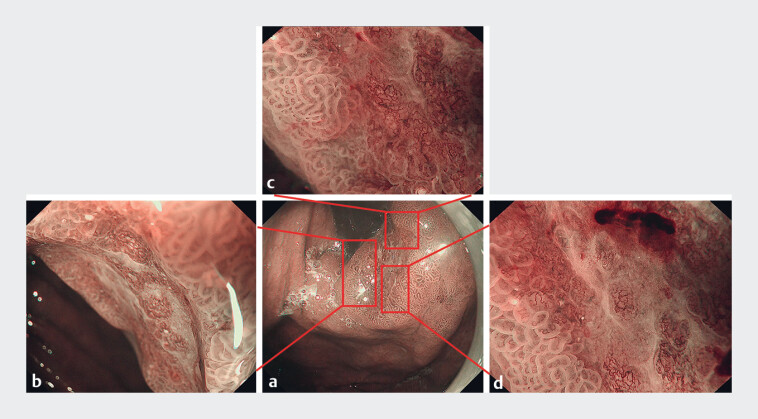
Detailed images of the 0-IIc lesion showing a clear demarcation line, irregular microvascular pattern with loop and irregular mesh pattern, irregular microsurface pattern with fused glands, unclear white zone, and white globe appearance.

An enhanced abdominal computed tomography scan showed no metastases and alpha fetoprotein levels (AFP) were normal. Endoscopic submucosal dissection of the two lesions was performed. Hematoxylin and eosin (H&E) staining of the resected 0-IIa lesion showed moderately differentiated tubular gastric adenocarcinoma with enteroblastic differentiation (GAED) in the mucous layer ( Fig. 4 ), with immunohistochemical analysis showing positivity for GPC-3 and SALL4 ( Fig. 5 ), while AFP staining was negative ( Video 1 ). The pathological diagnosis of this 0-IIa lesion was early gastric cancer, pT1a(M), ly(−), v(−), pR0, 20×6 mm (in 32×20 mm). The 0-IIc lesion showed moderately differentiated tubular gastric adenocarcinoma in the mucous layer. Endoscopic follow-up at 6 months revealed no signs of residual disease or recurrence.

**Fig. 4:**
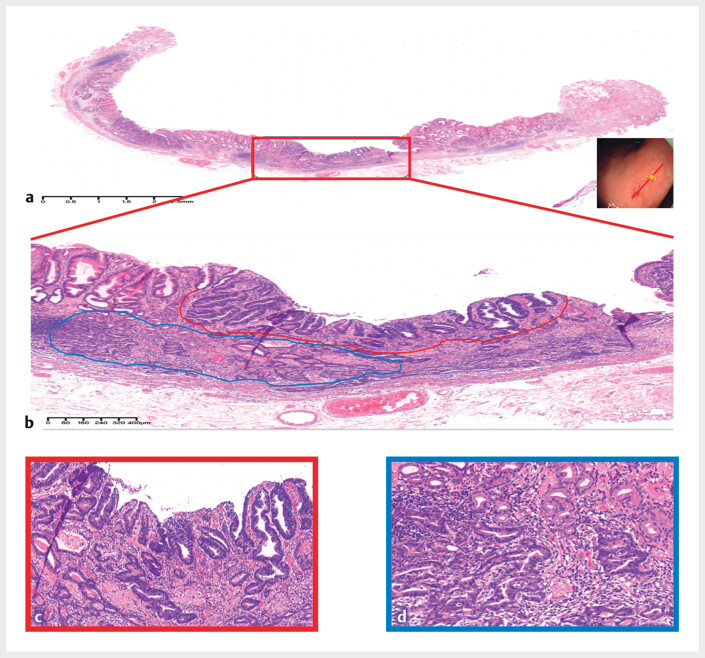
Hematoxylin and eosin (H&E)-stained sections of the 0-IIa lesion showing moderately differentiated tubular gastric adenocarcinoma with enteroblastic differentiation in the mucous layer.

**Fig. 5:**
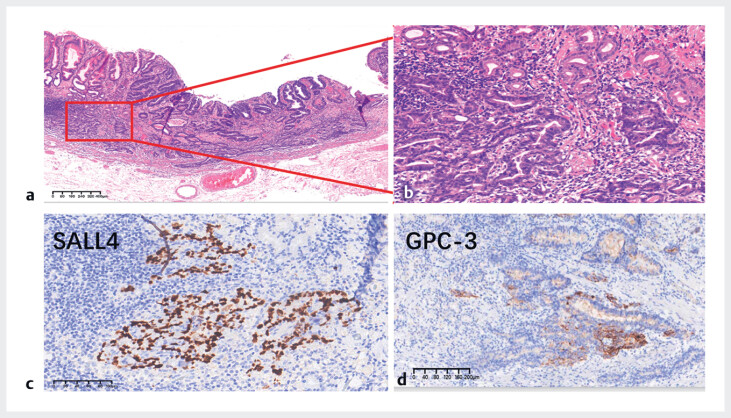
Sections of the 0-IIa lesion showing: **a, b** moderately differentiated tubular gastric adenocarcinoma cells with enteroblastic differentiation (GAED) on hematoxylin and eosin (H&E) staining; **c, d** positivity for SALL4 and GPC-3 on immunohistochemical staining, consistent with the diagnosis of GAED.

Endoscopic appearance, including on magnifying narrow-band imaging, of an early gastric adenocarcinoma with enteroblastic differentiation that was diagnosed synchronously with a conventional gastric adenocarcinoma.Video 1

The 0-IIa lesion with negative AFP may be explained by the early stage of GAED 1 . In GAED, the surface mucosal layer is covered by traditional tubular gastric adenocarcinoma. Tumors are generated from the deeper mucosal layer and invade the submucosal layer, producing the wide and elongated intervening part 2 . The eCura system aims to avoid unnecessary surgery 3 4 ; however, GAED is more aggressive than conventional gastric adenocarcinoma 5 . Therefore, a more precise eCura system combined with immunohistochemical analysis for GAED needs to be explored.

Endoscopy_UCTN_Code_TTT_1AO_2AG

## Notes

Endoscopy E-Videos https://eref.thieme.de/e-videos
*E-Videos* is an open access online section of the journal *Endoscopy* , reporting on interesting cases and new techniques in gastroenterological endoscopy. All papers include a high-quality video and are published with a Creative Commons CC-BY license. Endoscopy E-Videos qualify for HINARI discounts and waivers and eligibility is automatically checked during the submission process. We grant 100% waivers to articles whose corresponding authors are based in Group A countries and 50% waivers to those who are based in Group B countries as classified by Research4Life (see: https://www.research4life.org/access/eligibility/ ). This section has its own submission website at https://mc.manuscriptcentral.com/e-videos .
